# Trabecular metal monoblock versus modular tibial trays in total knee arthroplasty: meta-analysis of randomized control trials

**DOI:** 10.1007/s00264-022-05553-4

**Published:** 2022-08-29

**Authors:** Safa Abulhail, Shamsi Hameed, Maamoun Abousamhadaneh, Ghalib Al Haneedi, Mohamed Al Ateeq Aldosari

**Affiliations:** Orthopedic Department, Hamad Medical Corporation, Hamad General Hospital, 3050 Doha, Qatar

**Keywords:** Monoblock tibia, Cemented total knee, Modular tibia, Polyethylene, Trabecular metal

## Abstract

**Purpose:**

Total knee arthroplasty is one of the significantly evolving procedures with different knee designs available in the market. The continued development of these prosthesis resulted in improvement of the implant survivorship and patient satisfaction. This study is an RCT-based meta-analysis aimed to compare two designs of total knee replacement: the conventional modular and the monoblock trabecular metal tibial trays.

**Methods:**

This meta-analysis was performed by a literature review according to the PRISMA guidelines. A detailed search of the English literature was done using the PubMed, Medline, CINAHL, Cochrane, Embase, and Google Scholar databases. Only randomized control trials were included in the analysis after ensuring homogeneity. RevMan V.5.0.18.33 (The Cochrane Collaboration, Copenhagen, Denmark) was used to perform the meta-analysis. Extracted outcome measures were Knee Society score, Western Ontario and McMaster Universities Osteoarthritis Index (WOMAC) score, survivorship, complication rate, and radiostereographic analysis.

**Results:**

Seven randomized control trials with 635 patients were eligible for our analysis after they met our inclusion criteria. Three hundred twelve patients received monoblock tibias, and the other 323 patients received modular tibial trays during their total knee arthroplasty surgeries. There were statistically significant superiority of the modular knees in the functional Knee Society and WOMAC scores at five years (*P* = 0.003 and 0.05, respectively). The modular design was also more stable on RSA at two years (*P* < 0.0001).

**Conclusion:**

Modular and monoblock tibial trays are comparable knee designs with comparable survivorship and complication rates. However, the modular knees had better mid-term functional outcome and are more stable on radiostereographic analysis.

## Introduction

Total knee replacement surgery is a highly growing procedure that aimed to improve patients’ mobility and quality of life. While cemented modular knee designs have proven long-term durability and effectiveness [1, 2, 4], loosening of the tibial tray is one of the most common reasons for revision in total knee arthroplasty (TKA) [3]. Back-side wear of polyethylene inserts in TKA can produce polyethylene particles, leading to loosening of the tibial component. Loosening due to polyethylene debris could theoretically be reduced in tibial components of the monoblock polyethylene design, as there is no back-side wear [5, 7].

**Fig. 1:**
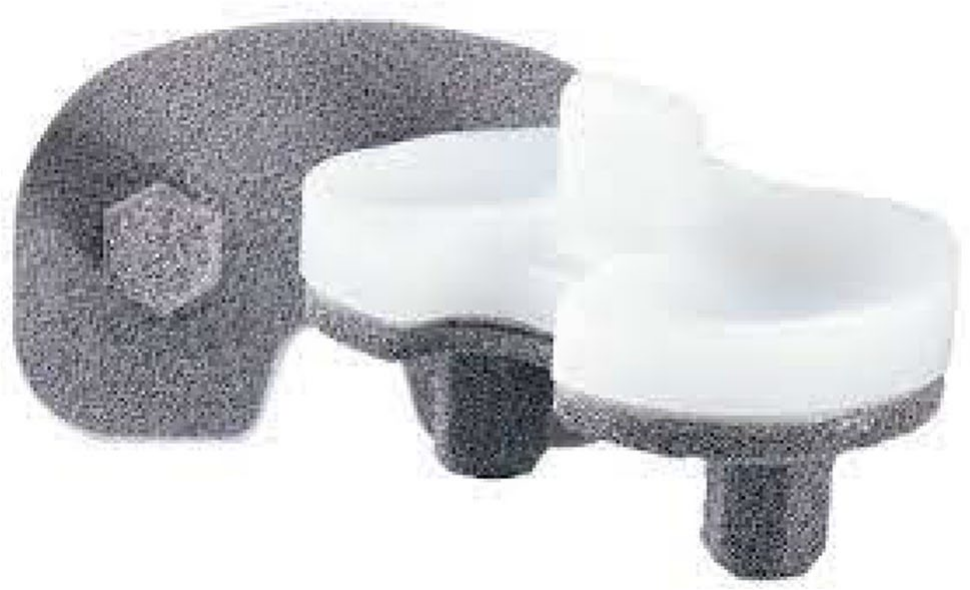
Demonstration of trabecular metal monoblock tibial trays

At the end of the 1990s, the trabecular metal tibial monoblock components were introduced as an alternative knee arthroplasty design [6]. As early results did not show any significant superiority compared to the standard designs, mid- and long-term studies confirmed good outcomes and survivorship of trabecular metal monoblock tibias [7, 9]. Additionally, using new porous metals in arthroplasty has improved bone-implant integration along with its favorable biomechanical properties. Furthermore, molding polyethylene into the metal would improve implant survivorship and reduce tray migration (Fig. 1). Nevertheless, all these advantages remain theoretical, and no strong evidence exists [9, 10].

This study aimed to provide the level of evidence comparing both designs by conducting a meta-analysis of seven published randomized control trials comparing the trabecular metal monoblock and modular tibial trays of total knee arthroplasties. Additionally, several clinical and radiological outcomes were pooled from the included studies, such as survivorship, complications, Knee Society score, WOMAC (The Western Ontario and McMaster Universities Osteoarthritis Index) score, and radiostereographic analysis (RSA).

## Materials and methods

**Fig. 2:**
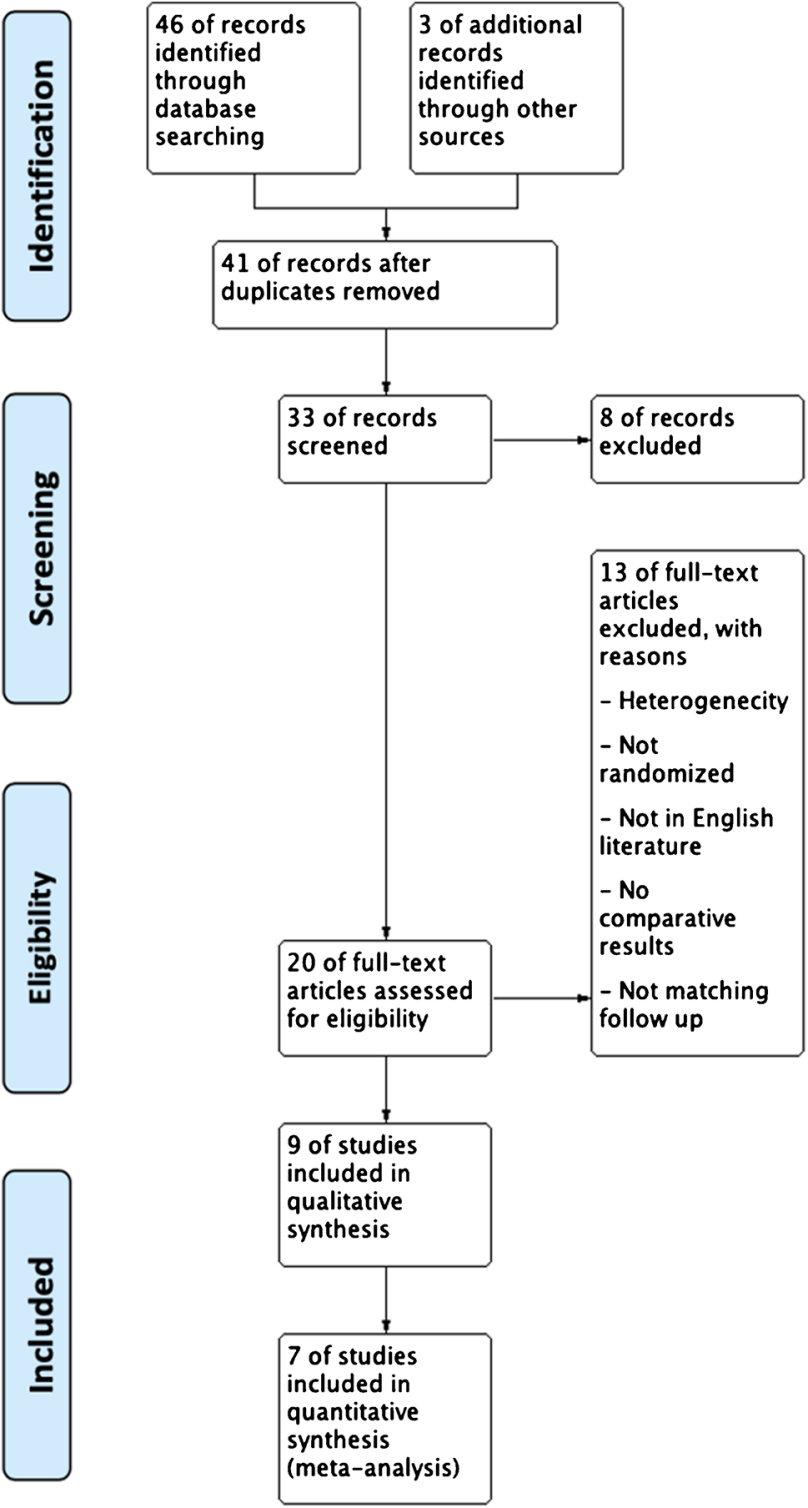
PRISMA checklist and algorithm

This meta-analysis was performed by a literature review and searched according to the Preferred Reporting Items for Systematic Reviews and Meta-Analyses (PRISMA) guidelines, with a PRISMA checklist and algorithm [22]. The algorithm is illustrated in Fig. 2.

A detailed search of the English literature using the keywords arthroplasty, total knee, tibial, trabecular metal monoblock, modular tibia, and polyethylene was performed using the PubMed, Medline, CINAHL, Cochrane, Embase, and Google Scholar databases. Only randomized control trials were included in the analysis after ensuring homogeneity. The search included all database entries that ended in May 2021.

Two investigators independently reviewed the literature and the full text of any article relevant to the research topic. We only included published randomized control trials comparing trabecular metal monoblock and modular tibial trays of total knee arthroplasties. Studies that were not in English or had no Level I evidence were excluded from the analysis.

The eligibility criteria for our search included (1) level I evidence, (2) a minimum of one year of follow-up, (3) published complete manuscript with available data, and (4) clear outcome measures with attached data presented as or can be transferred to mean and standard deviation values. Based on this, 20 full-text articles were reviewed in detail by the investigators, and only seven randomized control trials were eligible for analysis. The outcome measures that were common across the group of studies were as follows: (1) functional Knee Society score (KSS functional), (2) clinical Knee Society score (KSS clinical), (3) WOMAC functional score, (4) survivorship, (5) complication rates, and (6) RSA. The characteristics of the included studies are summarized in Table 1.

**Table 1:** Characteristics of the included studies

Study	Year of publication	Level of evidence	Number of knees	Modular	Monoblock	Follow up	Type of prosthesis	
							Modular	Monoblock
Henricson 11	2013	I	47	21	26	5 yrs	CR Cemented Selective patellar resurfacing NexGen (Zimmer)	CR Cementless TM Selective patellar resurfacing NexGen (Zimmer)
Fernandez-Fairen 15	2013	I	132	69	63	5 yrs	PCL-retaining Cemented modular Without patellar resurfacing stemmed modular tibial component (NexGen, Zimmer) — cemented	PCL-retaining Cementless monoblock Without patellar resurfacing TM monoblock tibial component (NexGen, Zimmer) — cementless
Pulido 16	2014	I	232	126	106	5 yrs	Cemented modular fluted tibial component (NexGen, Zimmer) PS patellar resurfacing	Cementless TM monoblock tibial component (NexGen, Zimmer) PS patellar resurfacing
Hampton 17	2020	I	77	41	36	2 yrs	CR Cemented tibia Without patellar resurfacing NexGen (Zimmer)	CR Uncemented tibia Without patellar resurfacing NexGen (Zimmer)
Andersen 13	2016	I	53	27	26	2 yrs	Uncemented Zimmer Nexgen trabecular metal CR Patellar resurfacing	Uncemented Zimmer Nexgen trabecular metal CR Patellar resurfacing
Wilson 12	2011	I	45	18	27	5 yrs	LPS cemented NexGen1 tibial component (Zimmer, Warsaw, IN, USA) PS patellar resurfacing	LPS monoblock uncemented NexGen1 tibial component (Zimmer, Warsaw, IN, USA) PS patellar resurfacing
Dunbar 14	2009	I	49	21	28	2 yrs	Cemented NexGen1 tibial component (Zimmer, Warsaw, IN, USA) PS patellar resurfacing	Monoblock uncemented NexGen1 tibial component (Zimmer, Warsaw, IN, USA) PS patellar resurfacing

The risk of bias was assessed by two authors who independently assessed the study methodologies using the Newcastle–Ottawa Scale [21] (Table 2). Studies were evaluated using the star scale for three variables: study population selection, comparability between the study groups, and the presented outcomes. Any disagreement between the reviewers was resolved by consensus. RevMan V.5.0.18.33 (The Cochrane Collaboration, Copenhagen, Denmark) was used to perform the meta-analysis. The mean and standard variations were extracted to represent continuous variables. Some studies presented their data with range, confidence interval, and/or first and third interquartile ranges. The Digitalizer software application was used in a study [17], whose data are presented as graphs. Validated formulas [18] were used to standardize the data into means and standard deviations, and when it was impossible to convert the values or in the presence of heterogeneity, the study was excluded [19, 20]. Dichotomous variables were analyzed using the relative risk with 95% confidence interval (CI). *I*^2^ was calculated as a measure of heterogeneity in the analysis, and the results were considered statistically significant when *P* < 0.05.

**Table 2:** Newcastle–Ottawa Scale

Study	Type	Selection				Comparability	Exposure/outcome			Total number of stars
Fernandez-Fairen	RCT	★	★	★	★	★★	★	★	★	9
Pulido	RCT	★	★	★	★	★★		★	★	8
Hampton	RCT	★	★	★	★	★★	★	★	★	9
Andersen	RCT	★	★	★	★	★★		★	★	8
Wilson	RCT	★	★	★	★	★★		★	★	8
Dunbar	RCT	★	★	★	★	★★			★	7
Henricson	RCT	★	★	★	★	★★		★	★	8

## Results

Overall, seven randomized controlled trials were eligible for our analysis after meeting our inclusion criteria. Overall, 635 patients were included in our meta-analysis; 312 patients received monoblock tibias, and the other 323 patients received modular tibial trays during TKA. All the included studies used the same prosthesis brand and randomized the study population according to the type of tibial implant of the same brand. All studies had equivalent randomized groups, and the populations were matched according to age and sex. There were some heterogeneities in the follow-up durations between the included studies; hence, we considered each outcome measure at close follow-up periods, except for the survivorship and the clinical Knee Society score, which were analyzed at the final follow-up that ranged between two and 15 years.

### Functional Knee Society score (KSS functional)

**Fig. 3:**
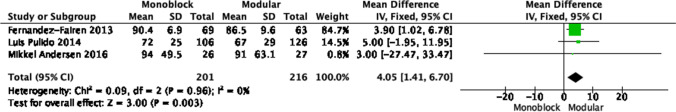
Forest plot of functional Knee Society score at the final follow-up between monoblock and modular tibias trays, CI confidence interval

The functional Knee Society score was reported in three RTC studies [13, 15, 16], and results were analyzed at the final assessment, which was at five years in two studies and two years in one study. One study only reported minimal differences favouring the monoblock tibial trays, whereas the other two reported no significant differences between the monoblock and modular tibias. Alternatively, our fixed model analysis reported a statistically better functional Knee Society score for the modular tibial trays, as shown in Fig. 3 (95% CI 1.41–6.7; *I*^2^ = 0%, *P* = 0.003).

### Clinical Knee Society score (KSS clinical)

**Fig. 4:**
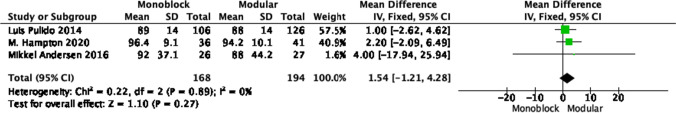
Forest plot of clinical Knee Society score at 2–5 years follow up between monoblock and modular tibias trays, CI confidence interval

The clinical Knee Society score was reported in three studies [13, 16, 17], and these studies reported the KSS outcome at different follow-up periods ranging from two to 15 years. Hampton et al. [17] reported their data at two, five and 15 years. Although they reported better clinical KSS of the monoblock design at 15 years in this particular study, they did not find any significant differences between the two groups at two and five years or in other studies [13, 16]. To avoid follow-up heterogeneity, we reported the clinical KSS at the final follow-up at two and five years in two studies [13, 16] and five years in one study. Our fixed-model analysis revealed no significant differences between the two knee designs at two to five years of follow-up, as shown in Fig. 4 (95% CI − 1.21 to 4.28; *I*^2^ = 0%, *P* = 0.27).

### The WOMAC functional score

**Fig. 5:**
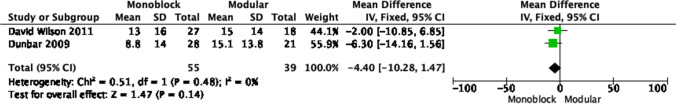
Forest plot of WOMAC score at 2 years follow up between monoblock and modular tibias trays, CI confidence interval

**Fig. 6:**
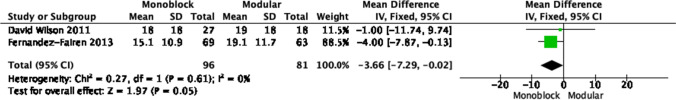
Forest plot of WOMAC score at 5 years follow up between monoblock and modular tibias trays, CI confidence interval

The WOMAC score has been reported in three studies [12, 14, 15]; a study [12] reported the outcome at two and five years, while the other two studies reported the outcome at one point, either two or five years. We extracted data at two and five years (Figs. 5 and 6). A study [15] reported slight superiority of the monoblock design at five years, while other studies did not show a significant difference in WOMAC scores. Our fixed model analysis revealed no differences between the two designs at two years (95% CI − 10.28 to 1.47; *I*^2^ = 0%, *P* = 0.14) and slight superiority of the modular knees at five years (95% CI − 7.29 to − 0.02; *I*^2^ = 0%, *P* = 0.05) when regarding WOMAC score.

### Survivorship

**Fig. 7:**
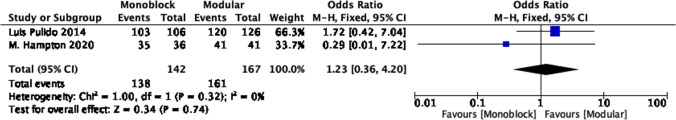
Forest plot of final survivorship follow-up between monoblock and modular tibias trays, CI confidence interval

Survivorship has been reported in two studies [16, 17]; one of the studies [16] reported survivorship at five years and the other one [17] at 15 years. Both studies reported comparable survivorships between the two designs at the final follow-up. We reported survivorship at the final follow-up, and our fixed model analysis (Fig. 7) revealed no significant differences between the two tibial designs (95% CI 0.36–4.2; *I*^2^ = 0%, *P* = 0.74).

### Complication rates

**Fig. 8:**
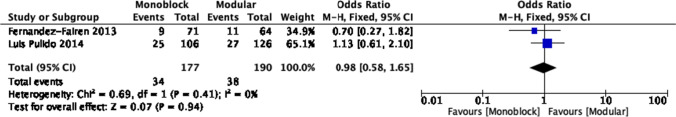
Forest plot of the complication rate at 5 years between monoblock and modular tibias trays, CI confidence interval

Complication rates were reported in two studies [15, 16], which reported the complication rates at five years, and none of them showed any superiority of either knee design. Furthermore, our meta-analysis (Fig. 8) did not show any differences in the complication rates between the two groups (95% CI 0.58–1.65; *I*^2^ = 0%, *P* = 0.94).

### RSA

**Fig. 9:**
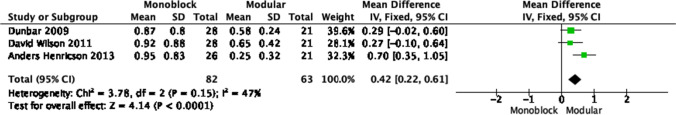
Forest plot of radiostereographic analysis (RSA) at 2 years between monoblock and modular tibias trays, CI confidence interval

The radiostereographic analysis (RSA) has been reported in four studies [11–14], and all of them have reported their results at two years. As RSA has multiple variables, we only reported the maximum total point motion (MTPM) in millimeters as it was mentioned in all studies. One study [13] was excluded as it compared cementless designs of both groups. The monoblock knee design was statistically more stable in this study [13] at two years, while the other three studies [11, 12, 14] did not report a statistically significant difference in the MTPM. Our fixed model analysis (Fig. 9) showed statistically significant stability of the modular cemented knee design compared to the cementless monoblock at two years of follow up (95% CI 0.22–0.61; *I*^2^ = 47%, *P* < 0.0001).

## Discussion

Total knee replacement is an orthopaedic procedure with a good long-term outcome [23, 26]. However, the longevity of the implanted prosthesis depends on multiple factors such as patient age, implant position, and fixation technique [22]. Previously, cemented modular knee arthroplasty was the most commonly used knee design, with good outcomes and survivorship. Nevertheless, osteolysis and implant loosening remain concerning when considering this design [24], and polyethylene back-side wear in the modular tibial component is a potential cause of osteolysis [20]. The introduction of the monoblock trabecular metal tibial component reduced the last two complications by enhancing bone-implant integration and omitting polyethylene wear [25–27].

This meta-analysis provided a high level of evidence comparing cemented modular and trabecular metal monoblock knee designs from different perspectives. This is the only RCT-based meta-analysis addressing this subject in the literature. In their meta-analysis, Bin Hu et al. [10] compared randomized and non-randomized data and heterogenic fixation methods and concluded that no significant differences were found between modular and monoblock tibias in TKAs; they attributed this to the variability of the included studies. Apart from Andersen et al. [13], who compared cementless modular to cementless monoblock tibial components, all the other six studies included in our meta-analysis compared cemented modular to cementless monoblock designs. Although this may have resulted in some heterogeneity in the analysis, the exclusion of Andersen et al. [13] from the analysis of KSS did not change the overall outcome; hence, we included it in the fixed model analysis.

Furthermore, Fernandez-Fairen et al. [15] was the only study that reported both WOMAC and functional KSS scores, and they reported the superiority of the cementless monoblock tibial trays at five years. Interestingly, our fixed model analysis revealed contradicting results for the WOMAC and functional KSS when the latter study was plotted with other papers. While WOMAC was significantly better in the monoblock knee design in our forest plot meta-analysis, functional KSS was superior to its modular counterpart. Additionally, this could be explained by the fact that outcome measures are inversely related, as the higher the KSS score, the better the result, and the lower the WOMAC score, the better the result. Hence, we concluded that the modular knee design was superior in functional KSS and WOMAC at five years of follow-up.

The follow-up periods were adjusted between the included studies, and most outcome measures were between two and five years. Hampton et al. [17], for instance, reported KSS at two, five and 15 years. However, they reported a superior clinical KSS score of the monoblock design at 15 years and similar scores at two and five years. Therefore, we selected the outcome at five years to plot with the other studies that reported their outcomes at the same period. However, survivorship was the only exception; two studies [16, 17] only reported survivorship at five and 15 years. Although this may lead to heterogeneity in the outcome, we considered survivorship in the final follow-up in our meta-analysis. With the follow up numbers given, we observe that most studies had almost similar outcome results at two and five years, and that is considered a mid-term follow up for a total knee surgery, and changes can be picked up at this period.

Furthermore, there were some outcome measures that we could not analyze, as they were not reported in more than one study. Pulido et al. [16], for instance, reported equivalent knee range of motion between the modular and monoblock tibial trays. However, our meta-analysis did not analyze that outcome, as no other study reported it. Furthermore, Fernandez-Fairen et al. [15], in their RCT, reported the need for the additional procedure at five years of follow-up, and they found that the modular knees received more additional procedures. However, this was not statistically significant.

Nevertheless, our analysis has several strengths, including its quality. It is the only level 1 meta-analysis in the literature reporting several outcome measures and some limitations. Heterogeneity and a short duration of follow-up were the most significant limitations. Most importantly, some of the included RCTs were more than 10 years old; hence, there were no adequate reports on the type of polyethylene used in these studies, which could lead to some uncertainty in the results. Further randomized trials are warranted in the future to elaborate further which design is better.

## Conclusion

Modular and monoblock tibial trays are viable options for TKA with almost equivalent survivorship and complication rates over five years. However, the modular tibial trays were significantly more stable, with lower maximal total point motion at two years; they also had significantly better functional outcomes at two to five years. Further long-term, high-quality studies are required to determine the superiority of either design.

## Data Availability

The raw data are available for any future need, and they can be obtained by contacting the corresponding author.

## Abbreviations

TKA: Total knee arthroplasty
RSA: Radiostereographic analysis
KSS functional: Functional Knee Society score
KSS clinical: Clinical Knee Society score
RR: Relative risk
CI: Confidence interval
